# Supplementary material for: Microbes translocation from oral cavity to nasopharyngeal carcinoma in patients
Source: Nat Commun. 2024 Feb 22;15:1645. doi: 10.1038/s41467-024-45518-2 (PMC10883945; doi:10.1038/s41467-024-45518-2)
Supplement: Supplementary file 1 — Supplementary Information File [file 41467_2024_45518_MOESM1_ESM.pdf]

Microbes translocation from oral cavity to nasopharyngeal carcinoma in patients

Author list:

Ying Liao[1,*], Yan-Xia Wu[1,*], Minzhong Tang[2], Yi-Wei Chen[1], Jin-Ru Xie[1], Yan Du[1], Tong-Min Wang[1], Yong-Qiao He[1], Wen-Qiong Xue[1], Xiao-Hui Zheng[1], Qiao-Yun Liu[3], Mei-Qi Zheng[1], Yi-Jing Jia[3], Xia-Ting Tong[3], Ting Zhou[1], Xi-Zhao Li[1], Da-Wei Yang[3], Hua Diao[3], Wei-Hua Jia[1,3,#]

Affiliations:

[1]State Key Laboratory of Oncology in South China, Collaborative Innovation Center for Cancer Medicine, Guangdong Key Laboratory of Nasopharyngeal Carcinoma Diagnosis and Therapy, Sun Yat-sen University Cancer Center, Guangzhou, China.

[2]Key Laboratory of Nasopharyngeal Carcinoma Molecular Epidemiology, Wuzhou Red Cross Hospital, Wuzhou, Guangxi, China.

[3]School of Public Health, Sun Yat-sen University, Guangzhou, China.

[*]These authors contribute equally to the work.

[#]Corresponding author:

Professor Wei-Hua Jia, M.D., Ph. D.

Sun Yat-sen University Cancer Center; State Key Laboratory of Oncology in South China; Collaborative Innovation Center for Cancer Medicine; Guangdong Key Laboratory of Nasopharyngeal Carcinoma Diagnosis and Therapy, Guangzhou 510060, China. Email: jiawh@sysucc.org.cn. Tel: 86-20-87342327.

Supplementary Information

Supplementary Fig. 1

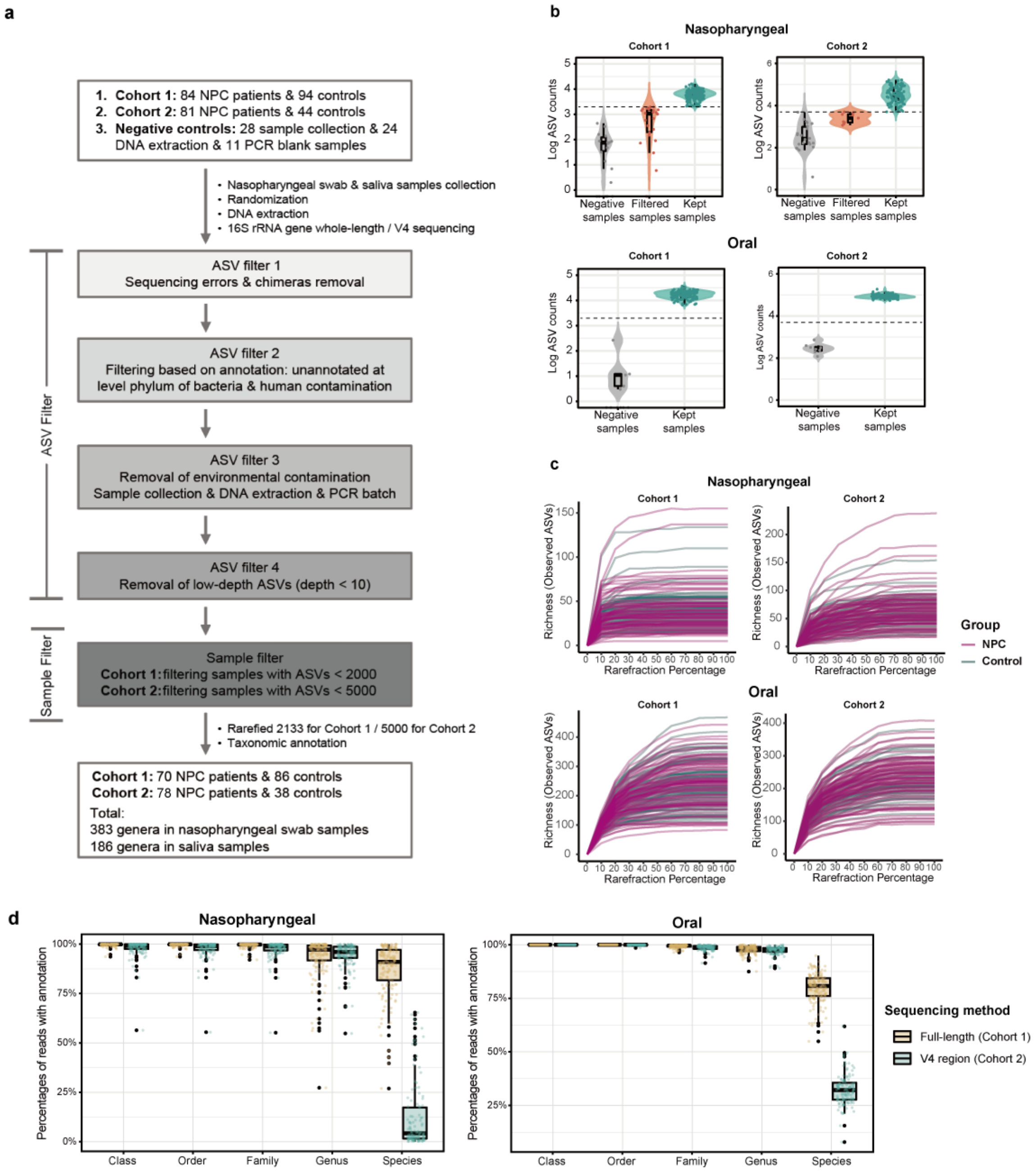

Supplementary Fig. 1 | Schematic diagram and quality control of 16S rRNA gene sequencing data.

(a) Schematic representation of the analysis pipeline applied to the sequencing data. (b) ASV counts per sample for 16S rRNA gene whole-length (Cohort 1) or V4-region

sequencing (Cohort 2) of microbial data. Negative samples included collection, DNA extraction and PCR blank negative samples. Filtered samples were excluded due to too few ASV counts. Kept samples were retained for subsequent analyses. Overlayed boxplots were presented with the median marked by thick black line, the interquartile range marked by the white bar, the range by the thin black line and outliners by the black dots. (c) The rarefaction curve generated based on 16S rRNA gene sequencing data of microbiota after quality control. An even sampling depth of 2133 and 5000 ASVs was used for subsequent analyses for Cohort 1 and Cohort 2, respectively. (d) Comparison between the full-length 16S data and V4-region data in terms of the proportion of reads that can be assigned to each classification level of taxonomies (Taxonomic level class, order, family, genus and species) for nasopharyngeal and oral microbiota. Boxplots were presented with the median marked by thick line, the interquartile range marked by the bar, the range by the thin line and outliners by dots. PCR, polymerase chain reaction; ASV, amplicon sequence variant. Source data are provided as a Source Data file.

Supplementary Fig. 2

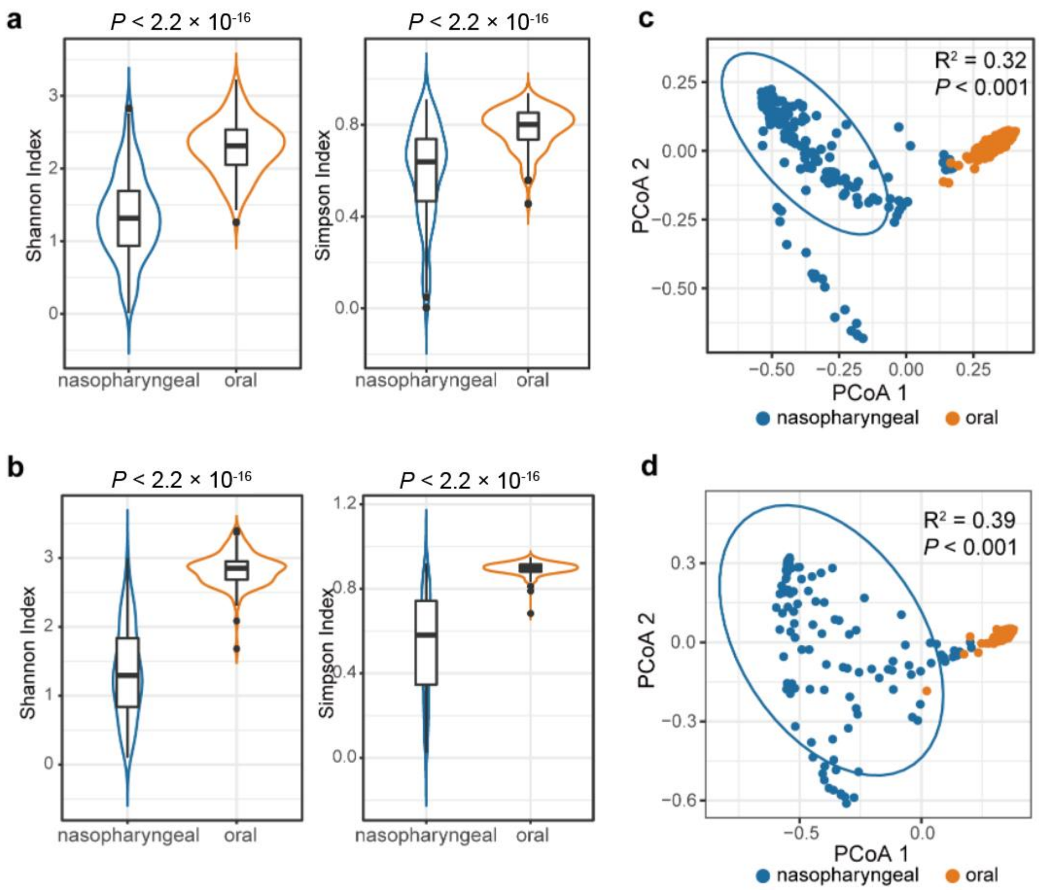

Supplementary Fig. 2 | Alpha and beta diversity differences between oral and nasopharyngeal microbiota.

(a, b) The violin plots of Shannon and Simpson indexes differences between nasopharyngeal and oral nasopharyngeal microbiota in Cohort 1 (a, N = 156) and Cohort 2 (b, N = 116). *P* values were determined by the Wilcoxon rank-sum test (two-sided). Overlayed boxplots were presented with the median marked by thick black line, the interquartile range marked by the white bar, the range by the thin black line and outliners by the black dots. (c, d) The PCoA plots based on Bray-Curtis dissimilarity distance show the compositional differences between nasopharyngeal and oral nasopharyngeal microbiota in Cohort 1 (c, N = 156) and Cohort2 (d, N = 116). R^2 and *P* values were determined by PERMANOVA analysis. Ellipses with 75% levels were shown. Source data are provided as a Source Data file.

Supplementary Fig. 3

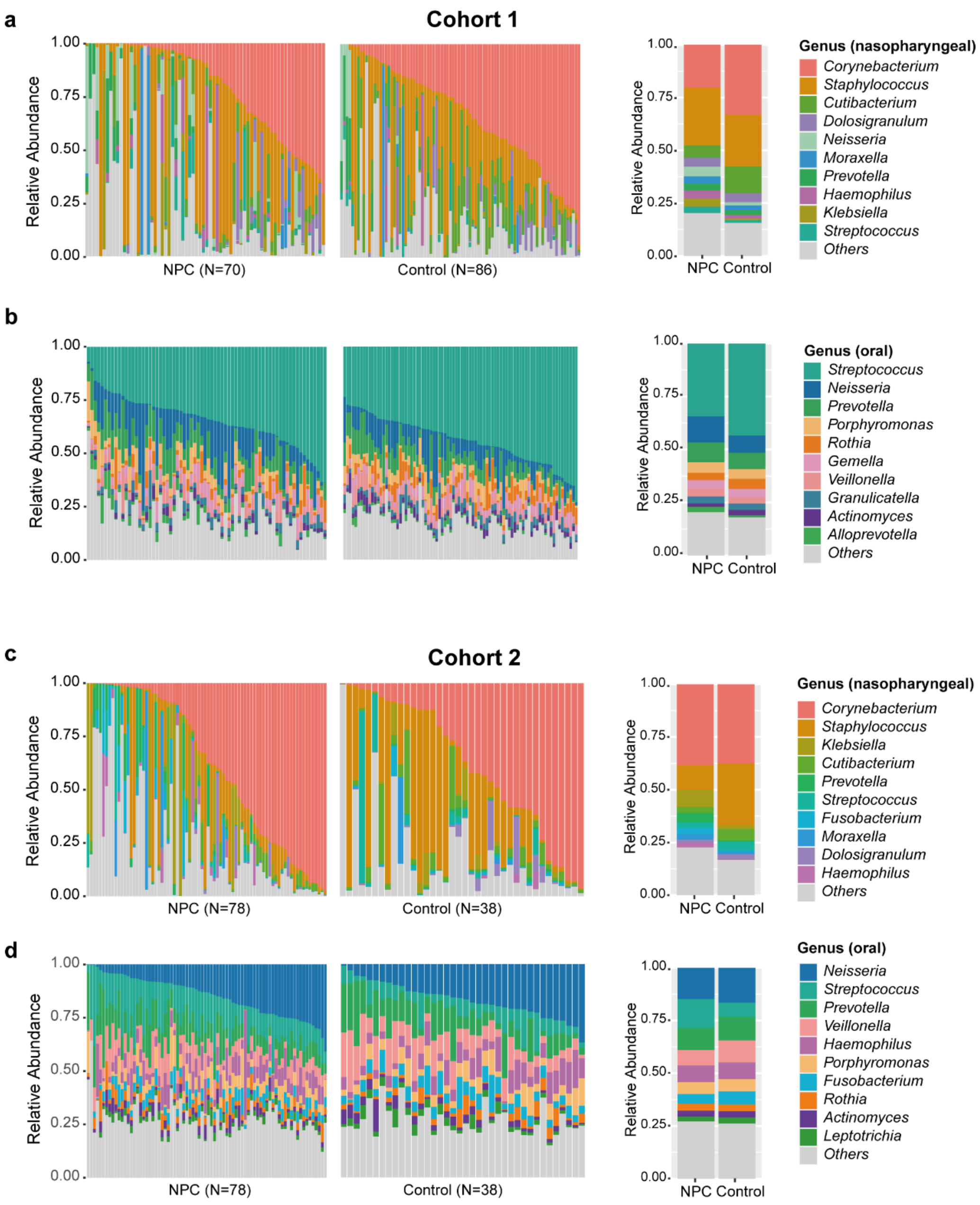

Supplementary Fig. 3 | Top 10 genera of nasopharyngeal and oral microbiota in two cohorts.

(a, b) The stacked bar plots of top 10 nasopharyngeal (a) and oral (b) genera of NPC and control groups in Cohort 1 (N = 156). (c, d) The stacked bar plots of top 10 nasopharyngeal (c) and oral (d) genera of NPC and control groups in Cohort 2 (N = 116). Source data are provided as a Source Data file.

Supplementary Fig. 4

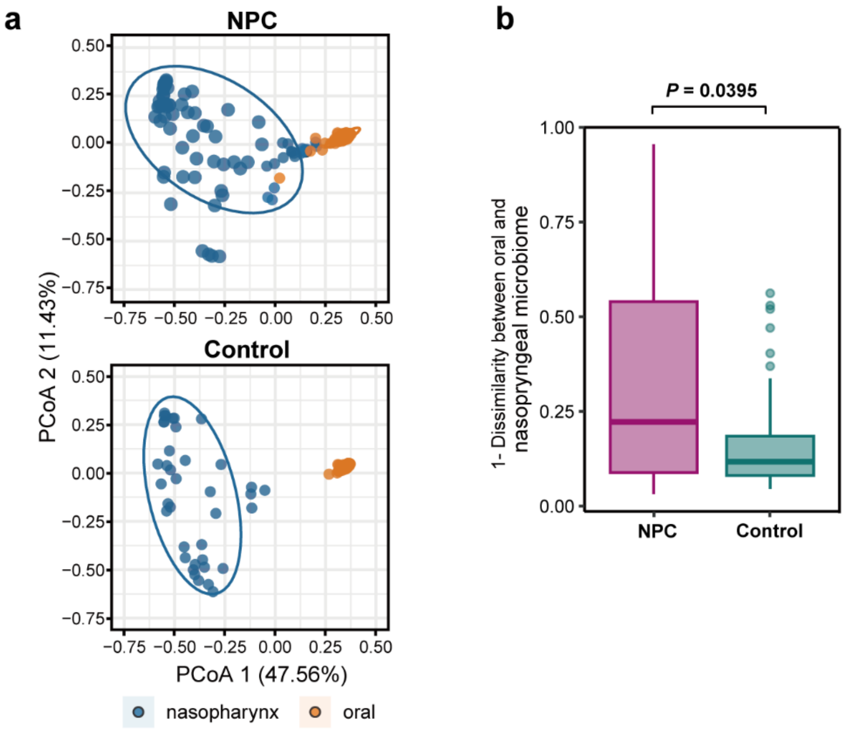

Supplementary Fig. 4 | The oral-nasopharyngeal similarity between NPC patients and controls in Cohort 2.

(a) The PCoA plots based on Bray-Curtis distance within NPC patients or controls. PCoA analysis was applied in combined NPC and control samples, and plots were presented separately on the same set of axes (N = 78 for NPC, and N = 38 for control). Ellipses with 75% levels were shown. (b) The oral-nasopharyngeal paired Bray-Curtis distance between NPC patients and controls. The *P* values between the two groups were determined by the Wilcoxon rank-sum test (two-sided). Boxplots were presented with the median marked by thick line, the interquartile range marked by the bar, the range by the thin line and outliners by dots (N = 116). Source data are provided as a Source Data file.

Supplementary Fig. 5

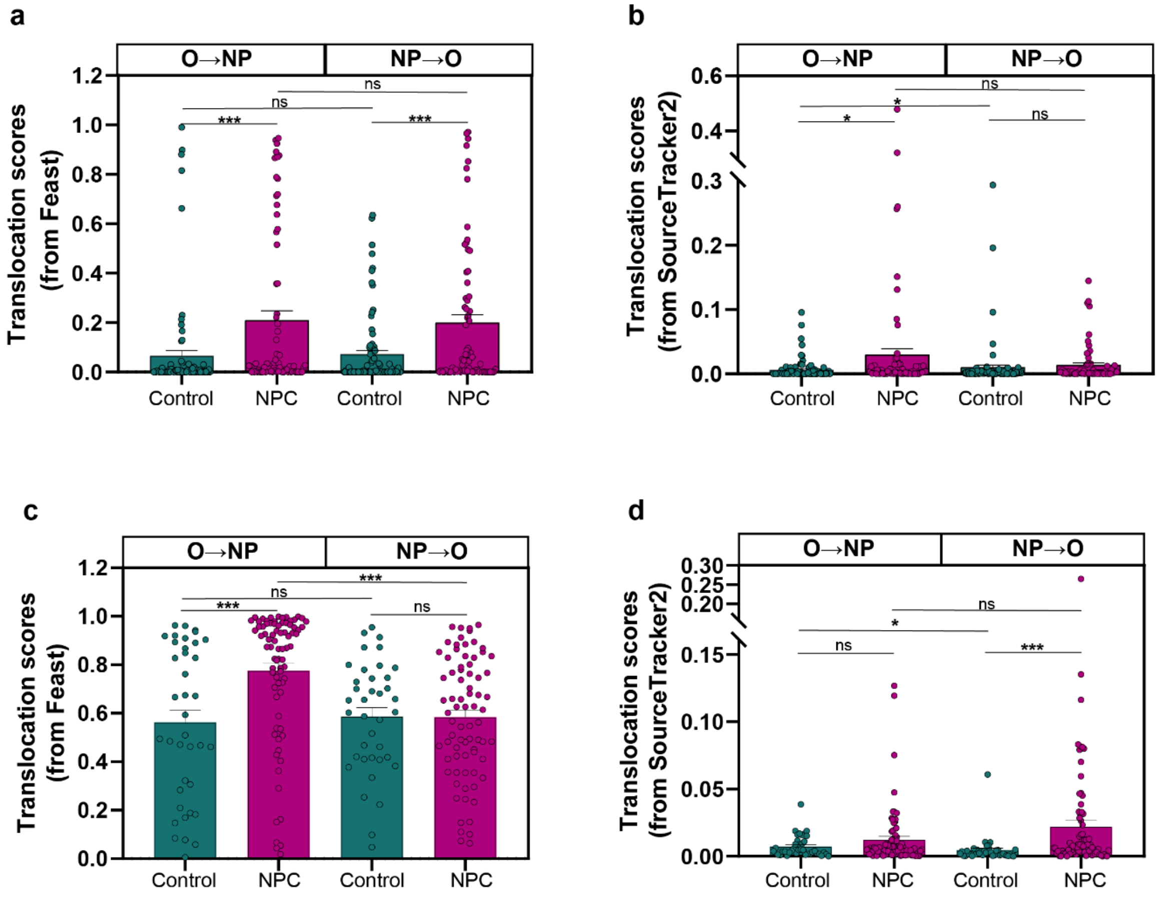

Supplementary Fig. 5 | The barplots showing the contribution of oral microbes to the nasopharyngeal community for each individual between NPC patients and controls. The translocation scores between NPC patients and controls in Cohort 1 (a - FEAST algorithm, b - SourceTracker2 algorithm, N = 156) and Cohort 2 (c - FEAST algorithm, d - SourceTracker2 algorithm, N = 116). *P* values were determined by Wilcoxon rank-sum test (two-sided). Data was shown in mean ± SEM. * $P < 0.05$, ** $P < 0.01$, *** $P < 0.001$, ns $P > 0.05$. Source data are provided as a Source Data file.

Supplementary Fig. 6

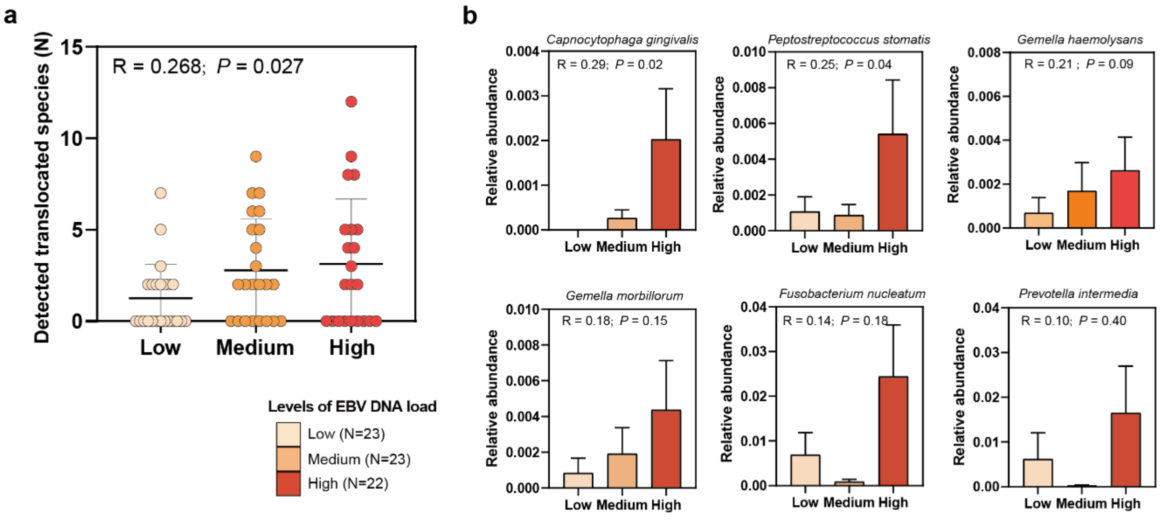

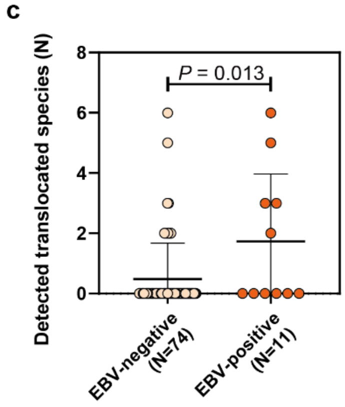

d

Species	Species detection rate in 74 EBV-negative controls N (%)	Species detection rate in 11 EBV-positive controls N (%)
Fusobacterium nucleatum	1 (1.4%)	2 (18.2%)
Peptostreptococcus stomatis	1 (1.4%)	1 (9.1%)
Prevotella intermedia	0 (0%)	0 (0%)
Gemella morbillorums	0 (0%)	0 (0%)
Capnocytophaga gingivalis	1 (1.4%)	0 (0%)
Gemella haemolysans	1 (1.4%)	0 (0%)

Supplementary Fig. 6 | The association between oral translocated microbes and EBV infection in nasopharynx in the NPC and control groups.

NPC patients were classified into three groups according to EBV load: low, medium and high groups (divided by 33% and 67% quartiles, N= 23, 23, 22 representatively). Controls were classified into two groups according to whether EBV DNA was able to detected (N = 74 for EBV-positive group, N = 11 for EBV-negative group). The association between the number of detected "NPC_{OtoNP}" microbes and EBV loads in the nasopharynx in the NPC (a, N = 68) and control (c, N = 85) groups. R and *P*-value were determined by Spearman's correlation analysis (two-sided). Data was shown in mean ± SD. (b) The correlation analyses between "NPC_{OtoNP}" microbes and EBV load in the NPC group. R and *P*-value were determined by Spearman's correlation analysis (two-sided, N = 68). Data was shown

in mean ± SEM. (d) The detection rate of NPC_{OtoNP} species in between EBV-negative and EBV positive individuals in the control group. Source data are provided as a Source Data file.

Supplementary Fig. 7

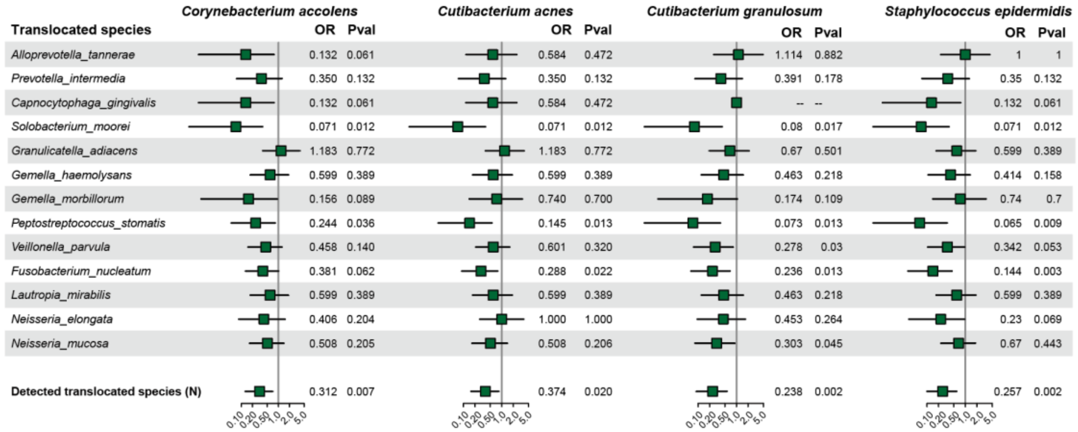

Supplementary Fig. 7 | OR estimates of nasopharyngeal commensals against translocated oral microbes.

ORs and *P* values were obtained from Logistic regression models (two-sided, N = 156). Independent variables were the abundance of commensals which were classified into two groups by the median abundances of each commensal. Dependent variables were the presence of the translocated oral microbes. The protective effects of commensals against the total number of detected translocated microbes were also estimated. Source data are provided as a Source Data file.

Supplementary Table 1 | Demographic characteristics of the participants in the cohorts.

	Cohort 1			Cohort 2		
	**NPC, N (%)**	**Control, N (%)**	***P* value**	**NPC, N (%)**	**Control, N (%)**	***P* value**
**No. participant**	**70**	**86**		**78**	**38**	
**Sex**			**0.003**			**0.824**
**Male**	**60 (85.7)**	**55 (64.0)**		**58 (74.4)**	**27 (71.1)**	
**Female**	**10 (14.3)**	**31 (36.0)**		**20 (25.6)**	**11 (28.9)**	
**Age, y**			**5.5×10^{-5}**			**0.117**
**Mean±SD**	**52.8±11.2**	**45.8±9.3**		**48.5±10.4**	**45.1±11.2**	
**Smoking**			**0.006**			**0.165**
**Yes**	**46 (65.7)**	**37 (43.0)**		**41 (52.6)**	**14 (36.8)**	
**No**	**24 (34.3)**	**48 (55.8)**		**37 (47.4)**	**23 (60.5)**	
**Drinking**			**1.000**			**0.800**
**Yes**	**15 (21.4)**	**18 (20.9)**		**16 (20.5)**	**6 (15.8)**	
**No**	**53 (75.7)**	**68 (79.1)**		**62 (79.5)**	**31 (81.6)**	
**Caries**			**0.065**			**2.0×10^{-5}**
**Yes**	**39 (55.7)**	**61 (70.9)**		**58 (74.4)**	**12 (31.6)**	
**No**	**31 (44.3)**	**25 (29.1)**		**19 (24.4)**	**24 (63.2)**	
**Oral/nasal Diseases**			**0.034**			**0.147**
**Yes**	**22 (31.4)**	**42 (48.8)**		**4 (5.13)**	**5 (13.2)**	
**No**	**48 (68.6)**	**44 (51.2)**		**72 (93.6)**	**32 (84.2)**	

P value was determined using *t*.test (two-sided) for continuous variables and Fisher's exact test (two-sided) for categorical variables; few missing values were contained in some variables.

Abbreviations: NPC, Nasopharyngeal carcinoma patients; SD, standard deviation.
