# Supplementary material for: Microbes translocation from oral cavity to nasopharyngeal carcinoma in patients
Source: Nat Commun. 2024 Feb 22;15:1645. doi: 10.1038/s41467-024-45518-2 (PMC10883945; doi:10.1038/s41467-024-45518-2)
Supplement: Supplementary file 3 — Description of Additional Supplementary Files [file 41467_2024_45518_MOESM3_ESM.pdf]

Description of Additional Supplementary Files

File Name: Supplementary Data 1

Description: Differential analysis of core taxonomies in nasopharyngeal microbiota between NPC patients and healthy controls in Cohort 1.

File Name: Supplementary Data 2

Description: Results of SparCC network analysis in the NPC group.

File Name: Supplementary Data 3

Description: Results of SparCC network analysis in the control group.

File Name: Supplementary Data 4

Description: Metadata of the participants in the culturomics study and their detected species in nasopharynx and oral cavity.

File Name: Supplementary Data 5

Description: Species and prevalence of microbes identified from the NPC tumors and normal tissues using meta-transcriptomic sequencing.

File Name: Supplementary Data 6

Description: Differentially expressed host genes identified in the tumor tissues between $OtoNP^{+}$ and $OtoNP^{-}$ NPC patients.

File Name: Supplementary Data 7

Description: Results from the enrichment analysis of Gene Ontology biological processes between $OtoNP^{+}$ and $OtoNP^{-}$ NPC patients.

File Name: Supplementary Data 8

Description: Results from the enrichment analysis of KEGG pathways between $OtoNP^{+}$ and $OtoNP^{-}$ NPC patients.

File Name: Supplementary Data 9

Description: The species significantly associated with nasopharyngeal EBV DNA loads.
