# Supplementary material for: Microbes translocation from oral cavity to nasopharyngeal carcinoma in patients
Source: Nat Commun. 2024 Feb 22;15:1645. doi: 10.1038/s41467-024-45518-2 (PMC10883945; doi:10.1038/s41467-024-45518-2)
Supplement: Supplementary file 13 — Reporting Summary [file 41467_2024_45518_MOESM13_ESM.pdf]

nature portfolio

Corresponding author(s): Wei-Hua Jia

Last updated by author(s): 2023-12-05

Reporting Summary

Nature Portfolio wishes to improve the reproducibility of the work that we publish. This form provides structure for consistency and transparency in reporting. For further information on Nature Portfolio policies, see our Editorial Policies and the Editorial Policy Checklist.

Statistics

For all statistical analyses, confirm that the following items are present in the figure legend, table legend, main text, or Methods section.

n/a	Confirmed	
☐	☒	The exact sample size (*n*) for each experimental group/condition, given as a discrete number and unit of measurement
☐	☒	A statement on whether measurements were taken from distinct samples or whether the same sample was measured repeatedly
☐	☒	The statistical test(s) used AND whether they are one- or two-sided<br>*Only common tests should be described solely by name; describe more complex techniques in the Methods section.*
☐	☒	A description of all covariates tested
☐	☒	A description of any assumptions or corrections, such as tests of normality and adjustment for multiple comparisons
☐	☒	A full description of the statistical parameters including central tendency (e.g. means) or other basic estimates (e.g. regression coefficient) AND variation (e.g. standard deviation) or associated estimates of uncertainty (e.g. confidence intervals)
☐	☒	For null hypothesis testing, the test statistic (e.g. F, t, r) with confidence intervals, effect sizes, degrees of freedom and P value noted<br>*Give P values as exact values whenever suitable.*
☒	☐	For Bayesian analysis, information on the choice of priors and Markov chain Monte Carlo settings
☒	☐	For hierarchical and complex designs, identification of the appropriate level for tests and full reporting of outcomes
☐	☒	Estimates of effect sizes (e.g. Cohen's *d*, Pearson's *r*), indicating how they were calculated

Our web collection on statistics for biologists contains articles on many of the points above.

Software and code

Policy information about availability of computer code

Data collection	No specific software was used for data collection.
Data analysis	All data analyses were conducted using publicly available tools. The following software were used in this study: SMRT Link software (v9.0.0); Lima (v2.0.0); R package: DADA2 (v1.22.0), Decontam (v1.10.0), ancombc (v1.0.5), FEAST (v0.1.0), ROCR (v1.0.11), edgeR (v3.36.0), ClusterProfilter (v4.2.2), GSVA (v1.42.0); KneadData (v0.10.0); Kraken2 (v2.1.1); Bracken (v2.5.0); Hisat2 (v2.1.1); htseq-count (v2.0.1); SourceTracker2 (v2.0.1); Mega (v7.0.21); Cytoscape(v3.9.0); SparCC (http://github.com/dlegor/SparCC); Trimmomatic (v0.39); Bowtie2 (v7.3.0); SPAdes (v3.15.5); PROKKA (v1.3); Roary (v3.11.2); InStrain (v1.0.0). The code developed for data analysis is available at https://doi.org/10.5281/zenodo.10083512.

For manuscripts utilizing custom algorithms or software that are central to the research but not yet described in published literature, software must be made available to editors and reviewers. We strongly encourage code deposition in a community repository (e.g. GitHub). See the Nature Portfolio guidelines for submitting code & software for further information.

Data

Policy information about availability of data

All manuscripts must include a data availability statement. This statement should provide the following information, where applicable:

- Accession codes, unique identifiers, or web links for publicly available datasets
- A description of any restrictions on data availability
- For clinical datasets or third party data, please ensure that the statement adheres to our policy

The metagenomics data generated in this study have been deposited in the National Center for Biotechnology Information (NCBI) under BioProject number

Research involving human participants, their data, or biological material

Policy information about studies with human participants or human data. See also policy information about sex, gender (identity/presentation), and sexual orientation and race, ethnicity and racism.

Reporting on sex and gender	This study includes both sexes. Sex was determined based on self-reporting. For the 16S rRNA gene sequencing, there were 218 males and 85 females included. After data quality control, 200 males and 72 females were eventually included in the study. Sex was included as a covariant for association analyses, and no sex-specific analysis was performed. Additionally, for the culturomics, there were 34 males and 14 females included.
Reporting on race, ethnicity, or other socially relevant groupings	Our study did not focus on social groups such as race and ethnicity.
Population characteristics	There were two cohorts for 16S rRNA gene sequencing. For Cohort 1, 70 nasopharyngeal carcinoma (NPC) patients and 86 controls were finally included. The mean age of the NPC group was 52.8 ± 11.2 years, and the mean age of control group was 45.8 ± 9.3 years. Males accounted for 86% of the NPC group and 64% of the control group. For Cohort 2, 78 NPC patients and 38 controls were finally included. The mean age of NPC group was 48.5 ± 10.4 years, and the mean age of the control group was 45.1 ± 11.2 years. Males accounted for 74% of the NPC group and 71% of the control group. In addition, for the other cohort used for culturomics, 34 NPC patients and 14 controls were included. Males accounted for 71% of the NPC group and 29% of the control group. The mean age of the NPC group was 49.4 ± 11.1 years, and the mean age of the control group was 47.6 ± 15.2 years. All NPC patients were pathologically confirmed.
Recruitment	Cohort 1 was collected at Wuzhou Red Cross Hospital in Wuzhou city, Guangxi province, China, from 2020-June to 2020-November; Cohort 2 was collected at Sun Yat-sen University in Guangzhou city, Guangdong Province, China, from 2018-June to 2019-Augest. In addition, the cohort used for culturomics was collected at Sun Yat-sen University in Guangzhou city, Guangdong Province, China from 2022-May to 2022-June and 2023-July to 2023-August. The patients who underwent nasopharyngeal endoscopy and pathologically diagnosed with nasopharyngeal carcinoma at both centers during this period were consecutively recruited in the study, and no subjective judgment was performed. We also recruited a group of healthy control subjects with the similar sex and age at both centers.
Ethics oversight	This study was approved by the ethics committee of Sun Yat-sen University Cancer Center. Informed consent was obtained from all study participants.

Note that full information on the approval of the study protocol must also be provided in the manuscript.

Field-specific reporting

Please select the one below that is the best fit for your research. If you are not sure, read the appropriate sections before making your selection.

☒ Life sciences ☐ Behavioural & social sciences ☐ Ecological, evolutionary & environmental sciences

For a reference copy of the document with all sections, see nature.com/documents/nr-reporting-summary-flat.pdf

Life sciences study design

All studies must disclose on these points even when the disclosure is negative.

Sample size	No statistical method was used to predetermine the sample size for this observational study. We included the NPC patients and controls with matched nasopharyngeal and oral microbial data collected at the same time. For this type of data, we included sufficient samples with reference to other microbial studies (e.g. PMID: 34234149, 36031410, 30883192).
Data exclusions	The data with insufficient sequencing depth and missing the pairing data were excluded.
Replication	Our main findings could be verified in two cohorts.
Randomization	Not applicable for this observational study.
Blinding	Not applicable for this observational study.

Reporting for specific materials, systems and methods

We require information from authors about some types of materials, experimental systems and methods used in many studies. Here, indicate whether each material, system or method listed is relevant to your study. If you are not sure if a list item applies to your research, read the appropriate section before selecting a response.

Materials & experimental systems

n/a	Involved in the study	
☒	☐	Antibodies
☒	☐	Eukaryotic cell lines
☒	☐	Palaeontology and archaeology
☒	☐	Animals and other organisms
☒	☐	Clinical data
☒	☐	Dual use research of concern
☒	☐	Plants

Methods

n/a	Involved in the study	
☒	☐	ChIP-seq
☒	☐	Flow cytometry
☒	☐	MRI-based neuroimaging
